# Office Visitors

**Published:** 1907-06

**Authors:** 


### OFFICE VISITORS.

He was a crank of cranks sure, from the land of cold bread, cold walls and hard tack. He bolted into the office, without any such trifling ceremony as a howdy do. That took time. He went right into biz. "Say! they tell me you're a pretty good dentist and I want to know if you can make a set of teeth like this one (taking out of his mouth a denture in which the teeth from molar to molar were bicuspids). You will note the peculiar construction of this set of teeth. They are made to grind. Every tooth having a broad grinding surface, but when in the mouth have the appearance of worn down teeth of a man of fifty or sixty—which age I am nearing. Now after exhaustive reading on the subject of food assimilation and its paradox dyspepsia or indigestion and I am visiting every resort of prominence in the world to get cured of the latter, I have made up my mind that thorough mastication is the greatest aid to digestion, which I could not perform with those swaying loose teeth, natural teeth and that horrible pyorrhoea I had. So I had my mouth cleared out and this set made according to my directions. I would not exchange them for all the patched up natural teeth in the world. I can grind infinitesimally with them. I notice though that several of the teeth are getting loose and are breaking next to the plate and for fear of an accident, I want a duplicate and I want it made within the next twelve hours." After more talk as to his ability to chew up chicken bones and reduce hard-tack to pulp, for he had soldiered and mined, and struck it rich at the latter, but had had tough food in the first and strenuous part of his career, we took the impression of his "cute yankee trick" and made for him a duplicate of his grinding teeth. It looked well in the mouth strange to say and pleased him as well as the original when inserted. "Take my advice doctor and make all your aged patients sets of teeth like mine and they will rise up and call you blessed and I don't care if you take out a patent on my invention." We thanked him for his advice and asked him to smile with us, but he smilingly refused—it was bad for his digestion to smile with spirit, but good when unfermented —so we parted smilingly—just so.

